# Aqueous humor proteomics analyzed by bioinformatics and machine learning in PDR cases versus controls

**DOI:** 10.1186/s12014-024-09481-w

**Published:** 2024-05-19

**Authors:** Tan Wang, Huan Chen, Ningning Li, Bao Zhang, Hanyi Min

**Affiliations:** 1 Department of Ophthalmology, Peking Union Medical College Hospital, Chinese Academy of Medical Sciences & Peking Union Medical College, No.1 Shuai Fu Yuan, Dongcheng District, Beijing, 100730 China; 2 Key Laboratory of Ocular Fundus Diseases, Chinese Academy of Medical Sciences & Peking Union Medical College, Beijing, 100730 China; 3 Operating Room, Peking Union Medical College Hospital, Chinese Academy of Medical Sciences & Peking Union Medical College, Beijing, 100730 China; 4 Animal Nutrition Institute, Sichuan Agricultural University, Chengdu, 611130 China; 5 Department of Ophthalmology, Aier Eye Hospital, Tianjin University, Nankai District, Fukang Road No.102, Tianjin, China

**Keywords:** Proliferative diabetic retinopathy, Proteomics, Machine learning, Biomarker, Bioinformatics analysis

## Abstract

**Background:**

To comprehend the complexities of pathophysiological mechanisms and molecular events that contribute to proliferative diabetic retinopathy (PDR) and evaluate the diagnostic value of aqueous humor (AH) in monitoring the onset of PDR.

**Methods:**

A cohort containing 16 PDR and 10 cataract patients and another validation cohort containing 8 PDR and 4 cataract patients were studied. AH was collected and subjected to proteomics analyses. Bioinformatics analysis and a machine learning-based pipeline called inference of biomolecular combinations with minimal bias were used to explore the functional relevance, hub proteins, and biomarkers.

**Results:**

Deep profiling of AH proteomes revealed several insights. First, the combination of SIAE, SEMA7A, GNS, and IGKV3D-15 and the combination of ATP6AP1, SPARCL1, and SERPINA7 could serve as surrogate protein biomarkers for monitoring PDR progression. Second, ALB, FN1, ACTB, SERPINA1, C3, and VTN acted as hub proteins in the profiling of AH proteomes. SERPINA1 was the protein with the highest correlation coefficient not only for BCVA but also for the duration of diabetes. Third, “Complement and coagulation cascades” was an important pathway for PDR development.

**Conclusions:**

AH proteomics provides stable and accurate biomarkers for early warning and diagnosis of PDR. This study provides a deep understanding of the molecular mechanisms of PDR and a rich resource for optimizing PDR management.

**Supplementary Information:**

The online version contains supplementary material available at 10.1186/s12014-024-09481-w.

## Background

The most frequent complication of diabetes and the major cause of blindness in the working-age population is diabetic retinopathy (DR), which has serious socioeconomic and quality-of-life effects [1, 2]. In the non-proliferative stage, DR begins with abnormal microvascular changes, characterized by increased vascular permeability, microaneurysms, and capillary closures. With the progression of the disease, neovascularization becomes evident, indicating the development of the proliferative stage. Proliferative diabetic retinopathy (PDR) is characterized by retinal neovascularization due to retinal ischemia. The formation of fibrovascular membranes (FVMs) and vitreous bleeding is caused by the overgrowth of the neovascular tufts toward the vitreous. In the severe stage of PDR, the FVMs can lead to tractional retinal detachment, resulting in devastating vision impairment.

In the pathogenesis of DR, hyperglycemia induces the production of superoxide in the mitochondria results in oxidative stress. This leads to multiple clinical hallmarks of DR, including pericyte apoptosis, basement membrane thickening, and mitochondrial dysfunction, resulting in a breakdown of blood retinal barrier (BRB) [3]. Then, the retina thickens and a leukocytosis increase occurs. As a result, white blood cells adhere to the endothelial cells lining blood vessels, which contributes to capillary plugging and vascular leakage [4]. As per the microvascular pathology, hypoperfusion due to pericyte loss damages the endothelium and leads to neovascularization, which compromises the BRB’s integrity. The process of neovascularization entails the formation of delicate and permeable blood vessels, which make vitreous hemorrhage more likely. Repeated hemorrhages cause fibrovascular scarring and gliosis, which when contracted, cause sight-threatening endpoints such as PDR and diabetic macular edema [3]. 

Classic treatments for PDR include intravitreal anti-vascular endothelial growth factor (VEGF) agents, laser photocoagulation, and vitreoretinal surgery [5]. Despite proper treatment received by many patients, it cannot prevent the advancement of the disease. Finding effective therapies in the early stages is difficult due to the intricacy of the pathophysiological mechanisms and molecular events associated with PDR.

The role of biomarkers in assessing health and planning medical interventions is vital. It is a powerful technology for discovering biomarkers using mass spectrometry (MS) based proteomics, but its use requires sophisticated bioinformatics to identify robust patterns [6]. With the advent of machine learning, biomarkers can now be discovered from proteomics data that outperform existing best-in-class assays [7]. Currently, a liquid biopsy can be used on the eye compartments, particularly the vitreous and aqueous, to study and phenotype intraocular diseases more directly [8, 9]. Aqueous humor (AH) protein concentration may be as clinically helpful as vitreous one in a variety of posterior segment diseases [10–13]. 

To fulfill these requirements and begin developing a precision medicine approach to PDR, AH proteomics has been applied to identify the differentially expressed proteins (DEPs). A strict bioinformatic analysis has been performed to obtain functional and pathway enrichment and hub proteins, and the inference of biomolecular combinations with minimal bias (iBM) [14] was utilized to screen ideal biomarker combinations to predict PDR. To validate the above results, an independent set of samples was subjected to parallel reaction monitoring (PRM) experiments to obtain quantitative information on the targeted proteins and the corresponding model validation. Taken together, our findings contributed to a better understanding of PDR and provided reliable biomarkers for early prediction, as well as nominated therapeutic targets for further treatment of the disease.

## Methods

### Subjects

Written informed consent was obtained from all enrolled patients in the study. The study was performed in compliance with tenets of the Declaration of Helsinki for biomedical research and was approved by the Ethics Review Committee of Peking Union Medical College Hospital (FW-HXKT2018103102421S2). The enrollment criteria of the PDR group were as follows: (1) clinical diagnosis of PDR [15]; (2) absence of other ocular diseases, pregnancy, or severe systemic conditions (except diabetes mellitus); and (3) absence of ocular treatment, such as photodynamic therapy, surgery, or intravitreal injection. In the control group, each patient should be diagnosed with senile cataract and scheduled for phacoemulsification cataract surgery for the insertion of an intraocular lens. They should have no history of other ocular diseases, prior intraocular treatment, or severe systemic conditions. All patients underwent pre-treatment ocular examinations, testing intraocular pressure (IOP), axial length, corneal endothelial cell counts, best-corrected visual acuity, B-ultrasonography, and biomicroscopy of anterior and posterior segments.

### Sample collection and preparation

The collection of AH was performed before treatment in both groups, regardless of whether it was pharmacological or surgical. AH was collected during microscope-aided surgery using a sterile 1-mL insulin injection syringe with a needle. The samples were collected in a 1.5-mL microcentrifuge tube and centrifuged at 13,000 rpm for 10 min at 4 °C, then transfer to a new 1.5-mL microcentrifuge tube and stored at -80 °C until subsequent analyses.

AH samples were sonicated three times on ice, using a high-intensity ultrasonic processor (Scientz, Ningbo, China), in lysis buffer (2 M Thiourea [Sigma-Aldrich, USA] + 7 M Urea [Amresco 0568-1Kg, USA] + 0.1% 3-[(3-Cholamidopropyl) dimethylammonio]-1-propanesulfonate [CHAPS] + protease inhibitors).

The remaining debris was removed by centrifugation at 12,000 g and 4 °C for 10 min. Furthermore, 10 µL of supernatant was collected and utilized by the Bradford Protein Assay Kit (Thermo 23,236, USA) for protein quantification. Proteins were then trypsin digested using the modified filter-aided sample preparation (FASP) technique [16, 17]. Briefly, lysate sample reduction was accomplished by incubating in 25 mM dithiothretitol (DTT) (Bio-Rad, USA) for 30 min at 60 °C, followed by 10 min of 50 mM iodoacetamide alkylation in the dark. After loading the samples onto a 10 kDa cutoff ultrafiltration membrane (Sartorius, Germany), they were incubated overnight at 37 °C with trypsin at a 1:50 enzyme-to-protein ratio. Following three 50 mM triethylammonium bicarbonate buffer (TEAB) (Sigma T7408, USA) rinses, the samples were treated with 10 min of spinning at 12,000 g. Peptide desalting was performed according to Ziptip C18 pipette tips in the manufacturer’s instructions.

After the C18 solid phase extraction column was activated and balanced with acetonitrile (CAN; Thermo A955-4, USA) and 2% ACN 0.1% formic acid (FA; Thermo A117-50, USA), the sample was loaded, followed by 10 times of pipetting, 2% ACN 0.1% FA desalination, and elution in 50% ACN 0.1% FA. The eluent was then collected into a rotary vacuum drier and refrigerated at -80 °C until use.

To build a data-independent acquisition (DIA) Spectral Library, dried peptides were subjected to resuspension in 0.1% FA and then collected and divided into samples with equal lysate quantities. The rest of the samples were used with the Biognosys iRT kit, including the preparation of a 10 × iRT buffer and the subsequent addition of it to each sample at 9:1.

### High-pH reversed-phase fractionation

Additional high-pH reversed-phase chromatographic separation of digest samples was performed. The reverse chromatography column (RIGOL, Beijing, China) was utilized for the separation of mixed peptides in a 30 µg digest sample. After the dissolution of peptides in mobile phase A (100 µL; 2% (v/v) ACN, 98% (v/v) ddH_2_O, pH 10), the mixture was spun down for 20 min at 14,000 g.

Then the mobile phase B (98% (v/v) acetonitrile, 2% (v/v) ddH_2_O, pH 10) was injected into the supernatants at 1 mL/min to achieve stepwise elution in the column. Mobile phase B step gradients were used to acquire individual 1.5-minute eluant fractions.

### MS acquisition

For MS analysis, we used an internally prepared analytical column (150 μm×150 mm, 1.9 μm) to evaluate each sample with a volume of 1 µg on an EASY-nLC1000 connected to an Orbitrap Fusion™ Tribrid™ MS instrument (Thermo Scientific). A binary solvent system, which was prepared by 0.1% FA in H_2_O (A) and 0.1% FA in ACN (B), was adopted, and the following linear gradient settings were used: 3–8% B/4 min, 8–22% B/65 min, 22–35% B/12 min, 35–90% B/4 min, 90% B/5 min.

Then the direct introduction of eluents into the MS instrument was performed using an EASY-Spray ion source, with the spray voltage and capillary temperature set at 2.3 kV and 320 °C, respectively. For data-dependent acquisition (DDA)-MS runs, the whole MS scanning ranged from 300 to 1400 m/z. The MS had a resolution of 60,000, with under 3-s top-speed mode for 15,000 resolution MS/MS scans. HCD had an isolation window and a normalized collision energy of 1.6 m/z and 32%, respectively. For DIA analyses, MS1 scans (automatic gain control (AGC) target 4e5 or 50 ms injection time) were performed from 300 to 1300 m/z, with DIA segmentation resolution of 30,000 (AGC target 5e5; for injection time). The collision energy was 32%, and the spectra were collected in profile mode.

### Identification and quantification of proteins

DIA data analyses adopted Biognosys’ Spectronaut pulsar programme and the ID picker algorithm [18]. The default software settings were employed for targeted data analyses, which included dynamic iRT for retention time prediction types with window-based correction factors. The enzyme specificity was configured to target the C-terminal of arginine and lysine residues, permitting a maximum of two missed cleavages during the database search. Peptide identification was performed with an allowed initial precursor mass deviation up to 10 ppm and an allowed fragment mass deviation 0.02 Da. The search criteria comprised carbamidomethylation of cysteine as a fixed modification, along with oxidation of methionine and acetylation at the protein N-terminus as variable modifications. The peptide-level false discovery rate (FDR) was set to 1% at both the protein and peptide precursor levels. Local mass calibration was utilized, along with limitless scrambled decoy generation. We also employed an MS2-level interference connection for fragment elimination based on interference signals while retaining ≥ 3 fragments for measurement. When conducting spectral library-based studies, RAW images were converted to the Spectronaut file format and calibrated according to the global spectral library’s retention time dimension. After that, the files were used for spectrum analysis without any further retention time-based recalibration.

Then, Proteome Discoverer 2.3 was used with default settings (Trypsin/P (Promega, V5111, USA), two missed cleavages). The fixed modification and the variable modifications in the search criteria were consistent with DIA data analyses. The mass tolerances for precursor and fragment ions were also set at 10 ppm and 0.02 Da, respectively [19]. DDA data searches used UniProt human (uniprot_human_73940_20190731_iRT.fasta) and Biognosys iRT peptides fasta (uploaded to the public repository) databases as references.

### Proteomic analyses

#### Statistical analysis of the quantitative data

After minimizing biases between experiments through median normalization, protein expression differences were then evaluated using a Student’s t-test. Statistically significant DEPs were defined using *p* adjust < 0.05 and fold-change (FC) cut-offs of |log_2_(FC)| > 0.58. Data normalization and identification of DEPs were performed in the ‘Wu Kong’ platform (URL: https://www.omicsolution.com/wkomics/main/) [20]. 

#### The enrichment analysis

To provide an intuitive and comprehensive visualization and direct comparison of DEPs data, heatmap was performed. The heatmap clustering analysis parameters were as follows: the scale direction is set to genes, gene clustering is performed using the complete method, distance calculation method is Euclidean, the callback function is set to pheatmap, and rows with completely identical expression values are removed.

Gene Ontology (GO) analysis has been used extensively to identify the characteristic biological attributes of genes, gene products, and sequences, including the biological process (BP), cell components (CC), and molecular function (MF) [21]. Kyoto Encyclopedia of Genes and Genomes (KEGG) analysis provides a comprehensive set of bio-interpretation of genomic sequences and protein interaction network information [22]. 

In this study, GO terms and KEGG pathway enrichment analyses were automatically completed and visualized using the clusterProfiler V3.14.0 [23], pathview V1.36.0 [24], and the Goplot V1.0.2 package [25] in the R software statistical analysis platform (significance was *p* < 0.05 and a q-value < 0.05).

#### PPI network construction and hub proteins identification

The protein-protein interaction (PPI) network of the DEPs was established using the Search Tool for the Retrieval of Interacting Genes (STRING) [26]. Cytoscape was used to build the visual network of molecular interactions with a combined score > 0.15 [27]. The molecular complex detection (MCODE) plugin was applied to detect closely correlated modules from the PPI network [28]. The most significant protein module of this PPI network was visualized and displayed through the MCODE plug-in. The filtering criteria were as follows: MCODE score > 5, node score cutoff = 0.2, degree cutoff = 2, k-score = 2, and max depth = 100. In addition, the degree, edge percolated component (EPC), betweenness, and maximum neighborhood component (MNC) algorithms were useful methods for selecting hub genes or proteins from PPI network [29]. Scores of the degree, EPC, betweenness, and MNC of all nodes of the PPI network were calculated via the CytoHubba plugin. The top 10 nodes with the highest degree, EPC, betweenness, and MNC scores were selected. Finally, to increase the reliability of hub proteins, their overlapping proteins were considered to be hub proteins related to PDR.

#### Machine learning-based inference of optimal biomolecular combinations

Identification of optimal biomolecular combinations using iBM that included mutual DEPs selection (MDS), candidate combination generation (CCG), and final combination prioritization (FCP) were carried out as previously described [14]. First, the true negative (TN), true positive (TP), false positive (FP), false negative (FN), sensitivity (Sn), and specificity (Sp) values were calculated. Then, 5-fold cross-validation was performed. The receiver operating characteristic (ROC) curve was illustrated and the area under curve (AUC) value was calculated based on Sn and 1-Sp scores. Third, the root mean squared error (RMSE) was calculated to estimate the prediction bias of a model.

CCG was adopted to select different sets of combinations with ≤ 5 proteins. Candidate combinations were randomly generated for the proteomic data. For each candidate combination, we randomly generated a training data set and a testing data set with a ratio of 4:1. The testing data set was only used to test the performance but not for training. The least absolute shrinkage and selection operator (LASSO, L1 regularization) penalty and the ridge regression (L2 regularization) penalty in penalized logistic regression (PLR) [30–32], were iteratively used to optimize the weight values of the 5 proteins. To simplify the combination, proteins with a weight of 0 in the model training results were deleted. All combinations with a total AUC equal to 1 were reserved for the optimal biomolecular combinations pool, respectively. The algorithm was implemented in Python 3.7 with Scikit-learn 0.22.1.

#### Validation study by PRM analysis

All the hub proteins determined above and the proteins of the top 25 combinations with the smallest root mean squared error (RMSE) values and AUC value of 1 were validated by PRM in independent samples.

First, the proteins were extracted, digested and mixed samples were prepared, and the full spectrum was scanned by the “label-free” method using the EASY-nLC1200 connected to the Orbitrap Q-Exactive HF mass spectrometer (Thermo, Scientific, USA). Second, the Proteome Discoverer 2.2 software was used to search the library. The search results were imported into Skyline(version 20.1.0.155) software [33] to obtain the target protein peptide information. Then, the PRM method can be established, and the obtained data were imported into Skyline software for quantification. The parameters of PRM were set as follows: the primary resolution was 12,000 (at 300–1400 m/z) with an automatic gain control (AGC) target value of 3e6, a maximum injection time of 80 ms, and a Normalized Collision Energy (NCE) of 27%; the secondary resolution was 15,000 with an AGC target value of 2e4, a maximum injection time of 19 ms. The mass tolerances for precursor and fragment ions were also set at 10 ppm and 0.02 Da, respectively.

The ROC curve analyses of the validated combinations were examined for PDR, and the AUC of each ROC curve was calculated.

#### Exploration for BCVA and early PDR-related proteins

To explore whether the hub proteins and the biomolecular combinations were associated with the best corrected visual acuity (BCVA) and the early occurrence of PDR, we used Spearman’s correlation analysis to evaluate the relations between the alteration of these proteins and the clinical parameters. A *p*-value of less than 0.05 was considered statistically significant.

## Results

### Study design and identification of DEPs

**Fig. 1:**
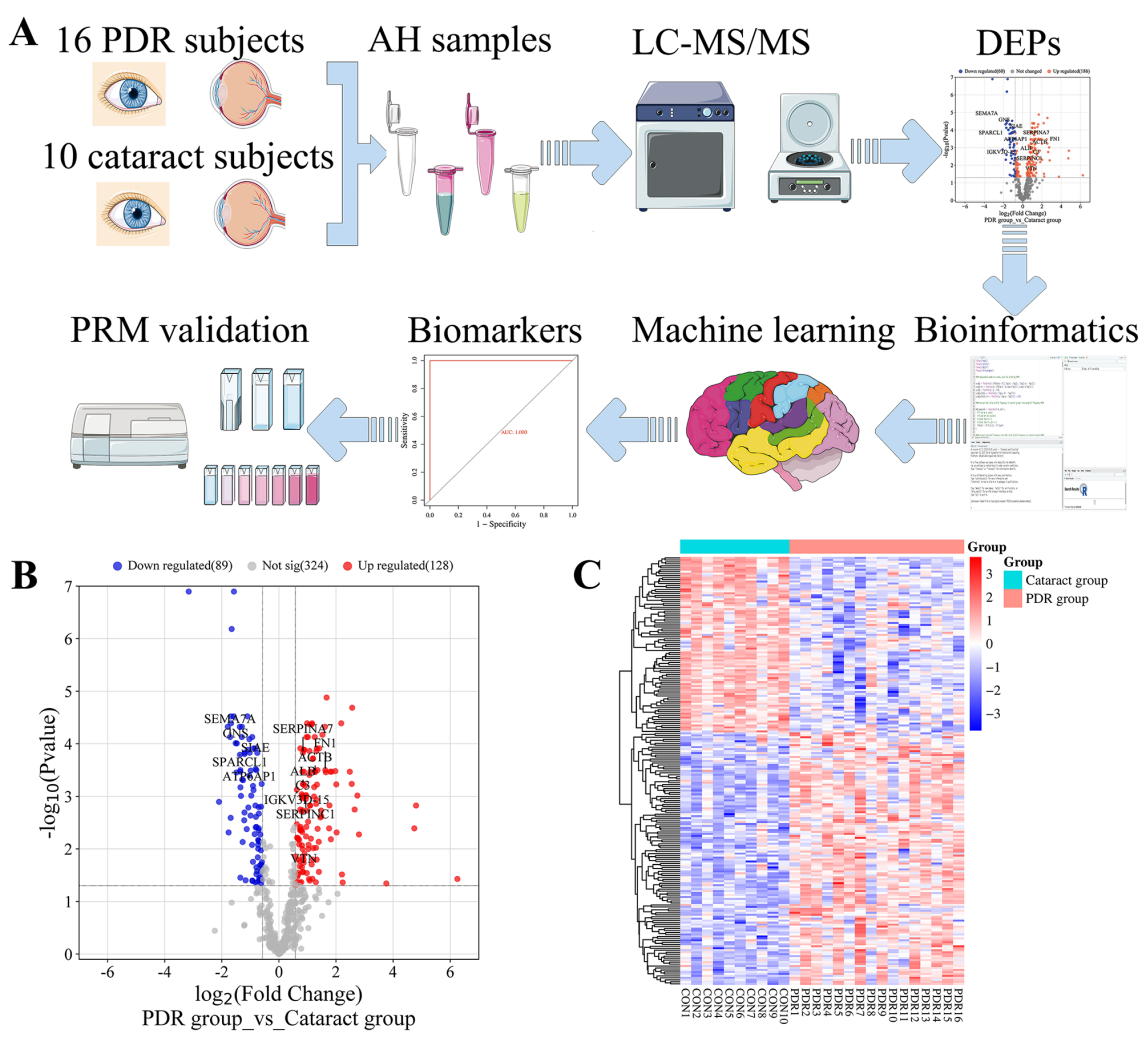
Study design and identification of differentially expressed proteins. **A**: Flow chart of the study. **B**: Valcano plot of differentially expressed proteins of two groups. **C**: Heatmap of quantification for the differentially expressed proteins. AH, aqueous humor; PDR, proliferative diabetic retinopathy; DEPs, differentially expressed proteins; LC-MS/MS, liquid chromatography-tandem mass spectrometry; PRM, parallel reaction monitoring

Figure 1 A shows the workflow of our study. Table 1 shows patients’ clinical features: 16 PDR patients and 10 cataract patients with corresponding mean ages of 57.5 ± 5.9 and 66.1 ± 12.6 years (*p* = 0.066). The two groups were statistically similar regarding gender, eye ratio, axial length, hypertension, or duration of hypertension; however, the difference in BCVA was statistically significant. All patients in the PDR group had diabetes, while all patients in the cataract group had no history of diabetes.

**Table 1:** Baseline characteristics of subjects included in the analysis^*^

Variables		PDR group (*n* = 16)	Cataract group (*n* = 10)	*p* ^†^
Age (years)		57.5 ± 5.9	66.1 ± 12.6	0.066^t^
Male gender (%)		7 (43.8)	4 (40.0)	1.000^F^
Right Eye (%)		11 (68.8)	5 (50.0)	0.425^F^
BCVA (LogMAR)		1.6 ± 1.0	0.2 ± 0.3	<0.001^U^
Axial length (mm)		22.7 ± 0.9	22.8 ± 1.0	0.891^t^
IOP (mmHg)		14.5 ± 2.9	15.4 ± 4.9	0.368^U^
Diabetes (%)		16 (100)	0 (0)	
Duration of diabetes (years)		14.8 ± 6.0	0	
Duration of PDR (Months)		6.5 ± 14.0	0	
Staging of PDR	IV	3 (18.8)	0	
	V	4 (25.0)	0	
	VI	9 (56.3)	0	
Hypertension (%)		9 (56.3)	7 (43.8)	0.420^P^
Duration of hypertension (years)		1.0 ± 4.0	0.0 ± 2.0	0.365^U^

^*^Quantitative data and qualitative data are expressed as the mean ± SD or median ± IQR and number of people (%), respectively; ^†^*p* values refer to independent Student’s t test, Mann‒Whitney U test, Pearson Chi-Square test and Fisher’s exact test used for exploring the differences in characteristics between two groups; t refers to independent Student’s t test; U refers to Mann‒Whitney U test; P refers to Pearson Chi-Square test; F refers to Fisher’s exact test

Large-scale LC-MS/MS analysis was performed on all gel bands, and a total of 874 unique proteins were retrieved (Table S1). After filtering by a 0.5 missing ratio in each group and filling by the k-Nearest Neighbor method (k = 5), 541 proteins common to all cases were further studied (Table S2), identifying 217 statistically significant DEPs; 128 were upregulated and 89 were downregulated (statistically significant DEPs). The DEPs are shown in the volcano plots and the heatmaps (Table S3; Fig. 1B and C).

### Characterization of DEPs and hub proteins in PDR patients

The annotations and functional enrichment analyses of GO biological processes and KEGG pathways were performed for DEPs. 744 significant GO terms and 30 KEGG pathways related to all 217 DEPs were discovered (Table S4 and S5). In addition, the number of DEPs based on GO function and KEGG pathways annotations were calculated.

**Fig. 2:**
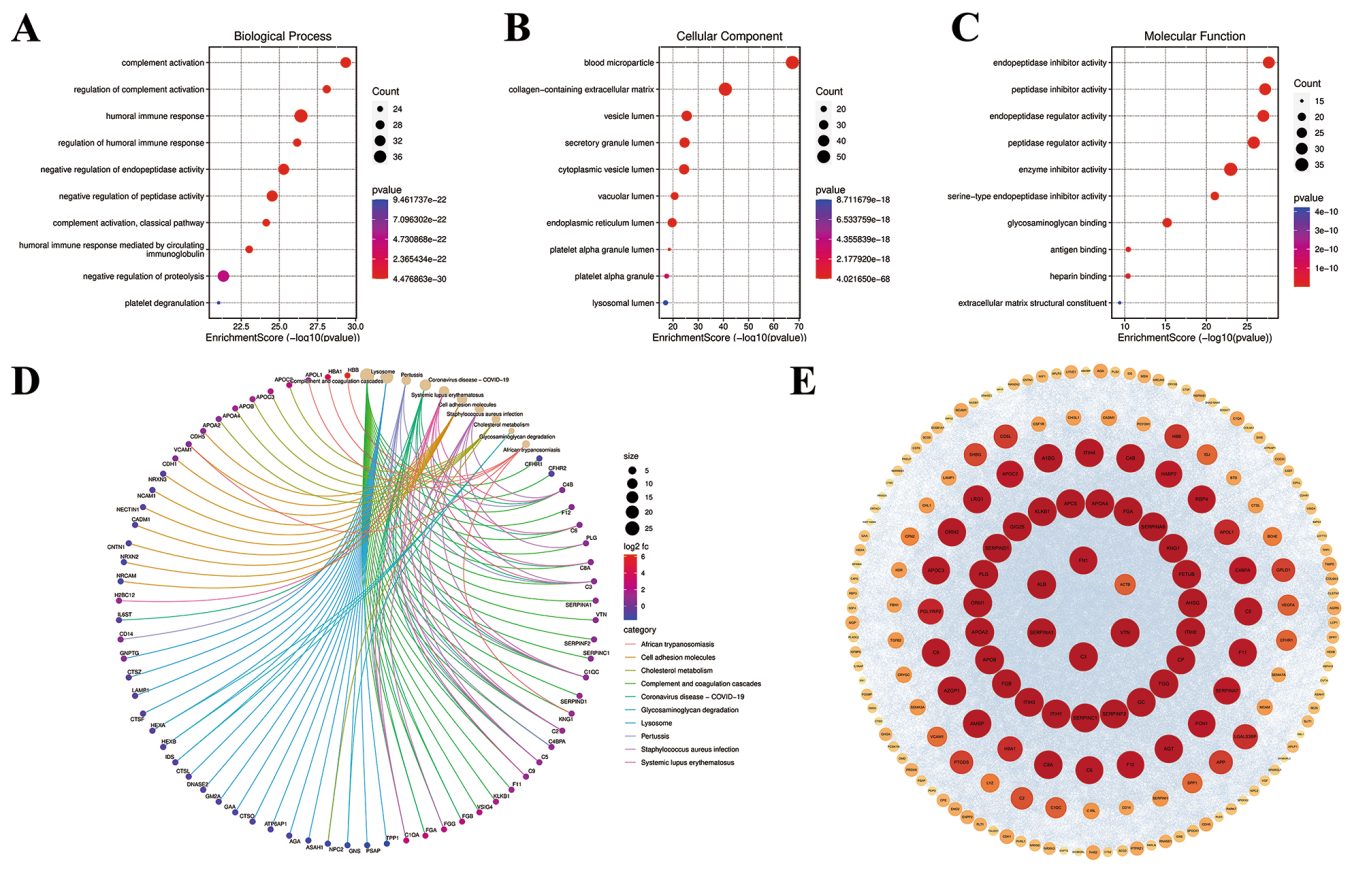
Characterization of differentially expressed proteins and hub proteins in proliferative diabetic retinopathy patients. **A**, **B**, and **C**: Gene Ontology (GO) enrichment analysis of differentially expressed proteins (**A**: molecular function. **B**: biological processes. **C**: cell composition). **D**: Kyoto Encyclopedia of Genes and Genomes (KEGG) pathway analysis of differentially expressed proteins. Proteins involved in the KEGG pathways are indicated by colored connecting lines. Symbols of differentially expressed proteins are presented on the left side of the graph. Symbols in red represent upregulated proteins, and blue represents downregulated proteins. The size refers to the representation of genes from the input list within those pathways. **E**: Protein-protein interaction network of differentially expressed proteins. The color and protein size were based on the molecular complex detection score; the higher the score, the darker the color and the larger the size. The 6 proteins in the center were the hub proteins we selected based on degree, edge percolated component, betweenness, and maximum neighborhood component scores

Enrichment analysis of DEPs was performed using Fisher’s exact test (*p* adjust < 0.05) to determine the overall functional enrichment characteristics of all DEPs and to find the most significantly enriched GO terms and KEGG pathways. The most significant enrichment of the BP term, MF term, and CC term was “complement activation” (*p* adjust < 0.001, 32 proteins), “endopeptidase inhibitor activity” (*p* adjust < 0.001, 31 proteins), and “blood microparticle” (*p* adjust < 0.001, 53 proteins), respectively (Fig. 2A, B, and C). “Complement and coagulation cascades” exhibited the most significant change in KEGG enrichment followed by “Lysosome” (*p* adjust < 0.001, Fig. 2D).

To better understand the relationship between DEPs, we utilized the STRING database for PPI analysis. The PPI can be classified as known interaction (curated databases and experimental determination from literatures), predicted interaction (gene-neighborhood, gene fusion, and gene co-occurrence), or others (text mining, co-expression, and protein homology). For detailed information, refer to Table S6. Among the 217 DEPs, 185 (85.3%) proteins were found to interact with other proteins (Fig. 4). The degree, EPC, betweenness, and MNC scores of DEPs were calculated using the CytoHubba plugin. We then selected the ten proteins with the highest scores in each algorithm and took the intersection of the four groups to improve the reliability of hub proteins. Finally, a total of six proteins (ALB, FN1, ACTB, SERPINA1, C3, and VTN) were considered to be hub proteins (Table 2; Fig. 2E). However, it should be noted that ACTB had a relatively low MCODE score compared to other hub proteins (Table 2; Fig. 2E).

**Table 2:** The betweenness, MNC, degree, EPC, and MCODE scores of hub proteins

Protein	Betweenness	MNC	Degree	EPC	MCODE
ALB	2013.7	143	143	28.7	41.9
FN1	1287.1	128	128	28.2	40.2
ACTB	1913.2	121	121	24.8	27.4
SERPINA1	620.1	110	110	28.4	41.9
C3	482.3	102	102	26.9	41.9
VTN	480.3	99	99	25.6	41.9

### Machine learning-based optimal biomolecular combinations in PDR

**Fig. 3:**
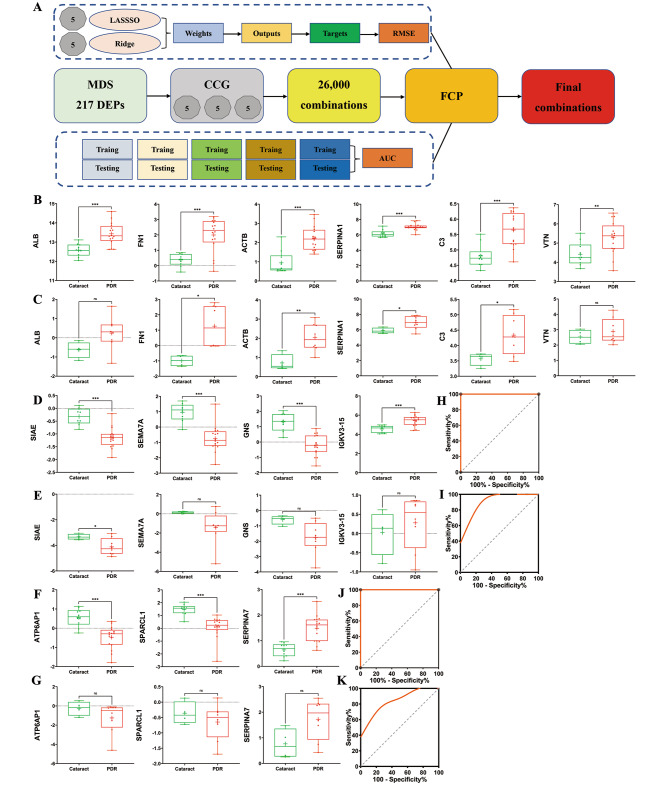
Machine learning-based optimal biomolecular combinations in proliferative diabetic retinopathy and comparison of two groups of quantification for the proteins based on data-independent acquisition (DIA) data and parallel reaction monitoring (PRM) data. *P* values comparing two groups in each graph were calculated using *p* adjust of Student’s t-test. **A**: The workflow of iBM, including MDS, CCG and FCP to prioritize candidate combinations with a maximal accuracy and a minimal bias from the 5-fold cross-validation. **B** and **C**: The quantification of hub proteins based on DIA data and PRM data, respectively. **D** and **E**: The quantification of proteins in the first biomarker combination based on DIA data and PRM data, respectively. **F** and **G**: The quantification of proteins in the second biomarker combination based on DIA data and PRM data, respectively. **H** and **I**: Receiver operating characteristic (ROC) curves of the first biomarker combination (SIAE, SEMA7A, GNS, and IGKV3D-15) predicting PDR based on data-independent acquisition (DIA) data and PRM data, respectively. **J** and **K**: ROC curves of the second biomarker combination (ATP6AP1, SPARCL1, and SERPINA7) based on DIA data and PRM data, respectively

Although 217 DEPs were identified, different molecules were altered to varying extents in PDR. The identification of optimal biomolecular combinations will not only be helpful for the accurate classification of different types of patients, but also provide useful information for uncovering the potential pathogenesis of PDR. Here, we utilized a pipeline named iBM, which consisted of three steps, including MDS, CCG to randomly select 26,000 combinations (including duplicates), and FCP to obtain the protein combination with a maximal accuracy and a minimal bias through the 5-fold cross-validation. The accuracy of a candidate model was evaluated by calculating the total AUC value, and we also computed the total RMSE to measure the prediction bias. In the step of FCP, a widely-used machine learning algorithm, PLR, was used for model training and parameter optimization (Fig. 3A). The combinations were determined for the proteomic DIA data (Table S7).

From the results, there were 22,423 protein combinations with a total AUC value of 1, although many combinations were repeated. With total RMSE values ranging from 0.198 to 6.665, the combination could perfectly distinguish PDR from cataract, with an AUC value of 1.

### Hub proteins and biomarkers validation by PRM

To further validate the hub proteins and the top 25 combinatorial biomarkers with the smallest RMSE values and AUC value of 1, we collected another 12 AH samples, including 4 samples from cataract patients and 8 samples from PDR patients. For the 25 combinatorial biomarkers, after removing of duplicate combinations, 15 combinatorial biomarkers remained (Table S8). In total, 43 proteins were selected for quantification by PRM, including ALB, FN1, ACTB, SERPINA1, C3, VTN, CUTA, ACTB, AGA, CTSZ, SPP1, CPVL, GM2A, SEZ6, ASAH1, SEMA3A, IGKV3D-20, SIAE, SEMA7A, GNS, IGKV3D-15, BTD, SCG3, IMPG1, OMD, PON1, TGFB2, IL6ST, HEXB, IGKV4-1, NPC2, RS1, PTPRZ1, CPE, TIMP2, IGLV1-51, SPOCK1, COCH, B4GAT1, JCHAIN, APLP2, FCGBP, and MAN2A2 hCG_32578.

It was revealed that hub proteins exhibited a similar up- or downregulation both in the DIA and PRM approaches (Fig. 3B and C; Table 3). Among the 15 biomarker combinations, 2 biomarker combinations can be completely verified by PRM results (Fig. 3D, E, F, and G; Table 3). The first biomarker combination consisted of SIAE, SEMA7A, GNS, and IGKV3D-15, with a total AUC and RMSE of 1 and 0.25 based on DIA data, respectively (Fig. 3H). The second biomarker combination consisted of ATP6AP1, SPARCL1, and SERPINA7, with a total AUC and RMSE of 1 and 0.29 based on DIA data, respectively (Fig. 3J). Based on the PRM data, the total AUC values for the two biomarker combinations were 0.938 and 0.875, respectively (Fig. 3I and K). Although individual molecules can achieve perfect accuracy on the current data, the combination of multiple molecules was undoubtedly important to reduce the prediction bias. The quantification of all the proteins by PRM is shown in Table S9 and Table S10.

**Table 3:** Comparison of targeted protein expression according to data-independent acquisition and parallel reaction monitoring methods

Protein	DIA_PDR vs. Cataract Log_2_ FC	DIA *p* adjust^†^	PRM_PDR vs. Cataract Log_2_ FC	PRM *p* adjust^†^	Consistency of DIA and PRM
ALB	0.846	<0.001	0.871	0.097	Yes
FN1	1.625	<0.001	2.256	0.027	Yes
ACTB	1.269	<0.001	1.330	0.027	Yes
SERPINA1	0.855	0.001	1.041	0.048	Yes
C3	0.845	<0.001	0.773	0.045	Yes
VTN	0.865	0.016	0.356	0.430	Yes
SIAE	-0.824	<0.001	-0.787	0.045	Yes
SEMA7A	-1.710	<0.001	0.959	0.100	No
GNS	-1.515	<0.001	-1.156	0.049	Yes
IGKV3D-15	0.628	<0.001	0.252	0.611	Yes
ATP6AP1	-1.035	<0.001	-1.003	0.242	Yes
SPARCL1	-1.365	<0.001	-0.288	0.405	Yes
SERPINA7	0.844	<0.001	0.959	0.100	Yes

^†^*p* values refer to independent Student’s t test

### Exploration for BCVA and early PDR-related proteins

**Fig. 4:**
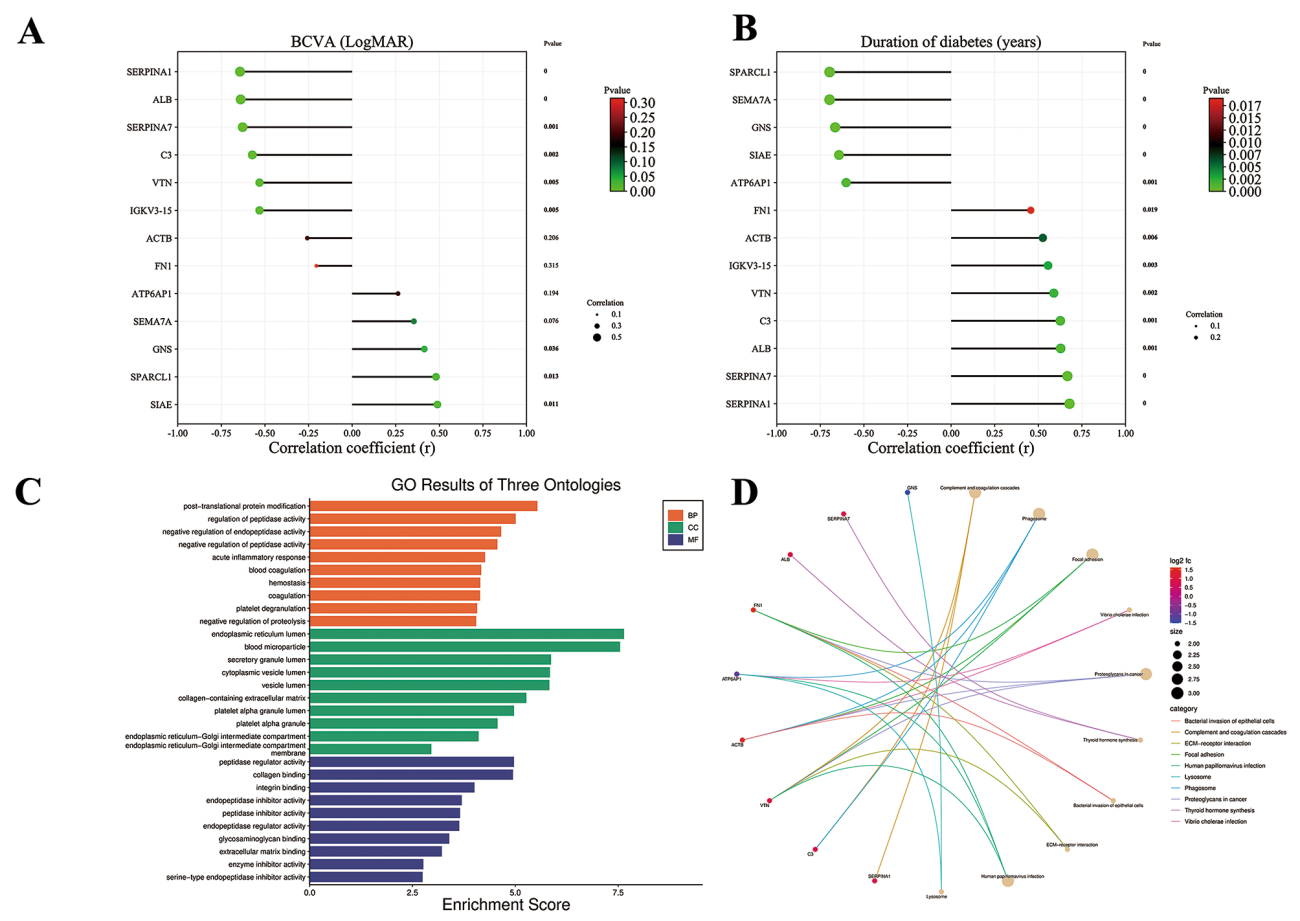
Exploration for best corrected visual acuity and early PDR related proteins and characterization of the hub and biomarker proteins. **A** and **B**: Correlation analysis of best corrected visual acuity and duration of diabetes with hub and biomarker proteins. **C**: Gene Ontology (GO) enrichment analysis of the hub and biomarker proteins. **D**: Kyoto Encyclopedia of Genes and Genomes (KEGG) pathway analysis of the hub and biomarker proteins. Proteins involved in the KEGG pathways are indicated by colored connecting lines. Symbols of differentially expressed proteins are presented on the left side of the graph. Symbols in red represent upregulated proteins, and blue represents downregulated proteins

Among the included patients, the BCVA of each individual varied greatly, and visual acuity was one of the most important factors affecting the quality of life. Additionally, the duration of diabetes also varied widely among PDR patients, implying differences in the rate at which their diabetic complications developed. Thus, Spearman’s correlation analysis was used to evaluate the degree of influence of the above proteins on these clinical parameters. As shown in Fig. 4A, SERPINA1, ALB, SERPINA7, C3, VTN, IGKV3-15, SIAE, SPARCL1, and GNS showed a significant correlation with BCVA (*p* < 0.001, *p* < 0.001, *p* = 0.001, *p* = 0.002, *p* = 0.005, *p* = 0.005, *p* = 0.011, *p* = 0.013, and *p* = 0.036, respectively). All the hub proteins (ALB, FN1, ACTB, SERPINA1, C3, and VTN) and the two biomarker combinations proteins (SIAE, SEMA7A, GNS, IGKV3D-15, ATP6AP1, SPARCL1, and SERPINA7) showed a significant correlation with the duration of diabetes (Fig. 4B, *p* = 0.001, *p* = 0.019, *p* = 0.006, *p *< 0.001, *p* = 0.001, *p* = 0.002, *p *< 0.001, *p *< 0.001, *p *< 0.001, *p* = 0.003, *p* = 0.001, *p *< 0.001, and *p *< 0.001, respectively). Interestingly, SERPINA1 had the highest correlation coefficient not only for BCVA (*r* = -0.643) but also for the duration of diabetes (*r* = 0.679). It can be regarded as a protective factor for vision and may also be able to delay the onset of PDR from diabetes.

### Characterization of the hub proteins and biomarker combinations

As the previous enrichment was performed on DEPs, the annotations and functional enrichment analyses of GO biological processes and KEGG pathways were also performed for the hub proteins and biomarker combinations. The most significant enrichment of the BP term, MF term, and CC term was “post-translational protein modification” (*p* adjust = 0.001, 5 proteins), “peptidase regulator activity” (*p* adjust < 0.001, 4 proteins), and “endoplasmic reticulum lumen” (*p* adjust < 0.001, 6 proteins), respectively (Fig. 4C). Interestingly, “Complement and coagulation cascades” also exhibited the most significant change in KEGG enrichment (*p* adjust = 0.007, Fig. 4D and S1).

## Discussion

Patients with PDR often experience unsatisfactory recovery of vision or even continued deterioration despite undergoing laser photocoagulation, repeated anti-VEGF therapy, and timely surgery. The mechanism of PDR remains unclear, making it crucial to gain a better understanding of the underlying processes driving PDR. To address this, we conducted a proteomic study to profile AH proteomic alterations in PDR and cataract patients, identifying a total of 217 DEPs between the two groups. Utilizing Fisher’s exact test, we found the most significant enrichment in the BP term, MF term, and CC term of GO analysis to be “complement activation”, “endopeptidase inhibitor activity”, and “blood microparticle”, respectively. “Complement and coagulation cascades” exhibited the most significant change in the enrichment of KEGG. Based on PPI analysis and four algorithms (degree, EPC, MCC, and MNC algorithms), the ten proteins of DEPs with the highest scores in each algorithm were selected, and the intersection of the results of the four groups was performed to determine six proteins (ALB, FN1, ACTB, SERPINA1, C3, and VTN) as hub proteins. The PRM validation of these hub proteins in another independent set of validation samples had similar protein expressions in the validation samples.

Furthermore, after utilizing an effective machine learning method based on DIA data and PRM data of the independent set of validation, we prioritized two optimal biomolecular combinations, each containing 4 and 3 proteins, respectively. These combinations showed accurate discrimination between PDR and cataract samples. The total AUC of each combination, based on DIA data, was 1. Based on PRM data, the total AUC of each combination was 0.938 and 0.875, respectively. These two combinations have the potential to be ideal diagnostic markers due to their high AUC values and lower RMSE than biomarkers of individual proteins. Correlation analysis revealed strong associations between the hub and biomarker proteins with BCVA and the duration of diabetes. Notably, SERPINA1 displayed the highest correlation coefficient not only for BCVA but also for the duration of diabetes. It can be considered a protective factor for vision and may also have the potential to delay the onset of PDR in patients with diabetes.

In this study, the enriched processes/pathways identified in PDR were largely in line with the previous proteomic profiling. Several independent studies on DR have also shown a GO enrichment of complement activation and KEGG pathway of complement and coagulation cascades. It has been suggested in some studies that the complement pathway plays a role in DR through the deposition of C3d and MAC complex in the choriocapillaries of DR eyes and the reduced level of glycosylphosphatidylinositol-anchored complement inhibitors, such as CD55 and CD59 in the walls of retinal vessels of DR eyes [34, 35]. Li et al [36] and Schori et al [37] performed a proteomic analysis on the vitreous humor of PDR patients. Both studies determined that the pathway of the complement and coagulation system was of great significance to PDR. There was also a proteomic study that grouped in a similar way as we did that also revealed an important role for the pathway of complement and coagulation cascades, although it lacked validation experiments. However, despite the detection of several complement proteins such as C3, CFI, CFB, C4A, C4B, C2, C4BPA, CFD, and CFH in PDR subjects in studies [38–41], their expression levels were highly variable among different studies and did not fully explain their exact involvement in DR pathology.

The hub proteins identified in this study may have an important role in the pathogenesis of PDR. ALB serves as a carrier protein for a variety of endogenous compounds, including hormones, fatty acids, and metabolites, as well as exogenous medicines. It also regulates colloid osmotic pressure and exhibits esterase-like activity with broad substrate specificity. However, Spranger et al. [42] reported that vitreous levels of ALB were 2.2-fold elevated in patients with PDR compared to controls, which is consistent with the results of the current study, both for the DIA data and the PRM data. The glycoprotein FN1 is found in the extracellular matrix and on the cell surface in soluble dimeric, dimeric, or multimeric forms. It plays a role in various cell adhesion and migratory activities, including metastasis, wound healing, blood clotting, and embryogenesis. VTN also stimulates cell adhesion and migration, inhibits the terminal cytolytic complement pathway’s ability to damage membranes, and binds to several serpin serine protease inhibitors. Additionally, it promotes the degradation of the extracellular matrix and participate in a wide range of other biological processes, including the regulation of the coagulation pathway, wound healing, and tissue remodeling. Casaroli Marano et al. [43] confirmed increased concentrations of intravitreous FN and ATN in PDR compared to normal samples, suggesting that FN and VTN play a key role in the structural arrangement of newly formed capillaries in PDR, and that receptor expression could be involved in events of endothelial cell adhesion and proliferation. ATN affects cell motility, structure, integrity, and intercellular signaling and is a crucial component of the contractile system. Increased ATN was detected in fibrovascular membranes with PDR by Cao et al. [44], suggesting it may play a positive role in the pericytes dropping out from microvessels. SERPINA1 is a serine protease inhibitor belonging to the serpin superfamily, and its targets include elastase, plasmin, thrombin, trypsin, chymotrypsin, and plasminogen activator. However, the role of SERPINA1 in the development of PDR has been poorly reported. By correlation analysis in the current study, we found that SERPINA1 had the highest correlation coefficient not only for BCVA but also for the duration of diabetes. This study may be the first to suggest its potential important role in the development of PDR. Follow-up studies can consider it as an important molecule for biological experiments or drug targets to explore its role in PDR. Complement component C3 plays a central role in the activation of the complement system. The GO and KEGG analyses in the current study revealed that the most significant enrichment of all DEPs was complement activation and complement and coagulation cascades, respectively, which has been corroborated in several previous studies [34–41]. Although ACTB had a relatively low MCODE score, externally validated data showed the potential value it may have in PDR. Few studies have reported its role in PDR. Thus, we plan to continue exploring it in future biology researches.

In recent years, machine learning-assisted proteomics or metabolomics biomarker screening methods have been applicated in DR. Sun et al. [45] used ultrahigh-performance liquid Q-Exactive mass spectrometry and the least absolute shrinkage and selection operator regularization logistic regression (LASSO-LR) based machine learning model to screen the plasma metabolome of 21 PDR and 53 non-PDR patients. They identified biomarkers consisting of four metabolites with an area under the ROC curve of 0.82. However, the predictive strength of the model in this study was still low, as no internal or external validation was performed, and RMSE was also not calculated to measure the prediction bias. In the current study, we randomly selected 26,000 combinations and performed 5-fold cross-validation to identify combinations with maximal accuracy and minimal bias. We evaluated the accuracy of each candidate model by calculating the total AUC value and also computed the total RMSE to measure the prediction bias. While AUC assesses discriminatory power, RMSE evaluates prediction accuracy. By considering both metrics together, we gain a comprehensive understanding of biomarker performance, allowing for more informed decisions regarding their criticality and relevance. We utilized the widely-used machine learning algorithm, PLR, for model training and parameter optimization. Additionally, to validate the protein expression of the ideal combinations and the stability of the model, PRM was conducted to detect the expression of the individual proteins in another independent sample set. As a result, we prioritized two optimal biomolecular combinations, each containing 4 and 3 proteins, respectively. Both combinations accurately distinguished the samples of PDR from cataract. Based on DIA data, each combination had a total AUC of was 1. Based on PRM data, the total AUC of each combination was 0.938 and 0.875, respectively. Thus, these two combinations hold potential to serve as ideal diagnostic markers.

There are several limitations in our study. First, for medical research ethical reasons, AH samples from healthy individuals could not be collected, so cataract patients were included in the control group. Another possible drawback of this work is that the sample size in this study was relatively small. Third, two optimal biomolecular combinations were computationally prioritized by iBM and PRM validation, in which total RMSE values were calculated to estimate and reduce the prediction bias. However, overfitting may not be entirely avoided for the finally determined models. Furthermore, it has been demonstrated that results based on proteomics varied greatly between different research studies [46–48]. There are several variables that could significantly affect the outcomes, including cohorts from different nations or locations, variations in age, sex, body mass index (BMI), and physical conditions, as well as various sample preparation techniques and data analysis platforms [49]. Therefore, even though PRM was carried out to verify the results, we believe that enrolling more samples, perhaps from various centers, would be more beneficial to further verify the results. Finally, more research is required to determine the detailed functions of the hub proteins and whether they could be used as clinically effective therapeutic targets.

In summary, our findings provided a highly valuable proteomic data resource for the research community to better understand PDR. We identified several proteins specifically altered in PDR patients, shedding light on the pathogenesis of PDR, and providing potential biomarkers for diagnosis of the disease and target molecules worth investigating.

### Declaration of generative AI and AI-assisted technologies in the writing process

During the preparation of this work, the authors used ChatGPT in order to improve language and readability. After using this tool, the authors reviewed and edited the content as needed and take full responsibility for the content of the publication.

### Electronic supplementary material

Below is the link to the electronic supplementary material.

Supplementary Material 1

Supplementary Material 2

## Data Availability

The datasets generated during the current study are available in the supplemental tables. Any additional information required to reanalyze the data reported in this paper is available from the lead contact upon request.

## Declarations

### Note

No portion of the contents of this paper has been published previously.
