# Supplementary figures and images for: Aqueous humor proteomics analyzed by bioinformatics and machine learning in PDR cases versus controls
Source: Clin Proteomics. 2024 May 19;21:36. doi: 10.1186/s12014-024-09481-w (PMC11103871; doi:10.1186/s12014-024-09481-w)

**Supplementary Information**

**Figure S1.**


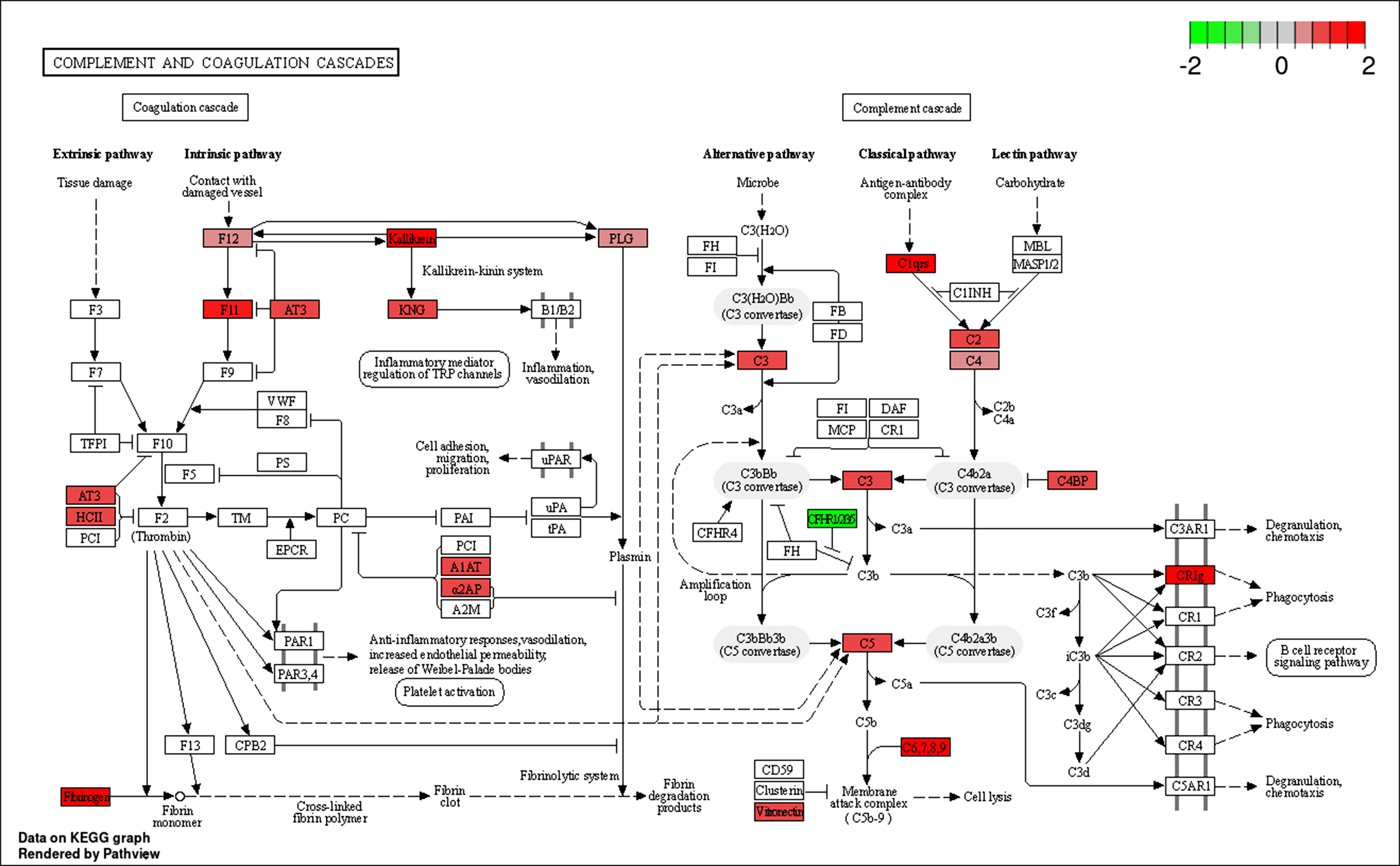

Supplement: Supplementary file 2 — Supplementary Material 2 [file 12014_2024_9481_MOESM2_ESM.docx]
